# Non-Exonuclease Domain *POLE* Mutations Associated with Immunotherapy Benefit

**DOI:** 10.1093/oncolo/oyac017

**Published:** 2022-02-28

**Authors:** Sharlene Dong, Heba Zakaria, David Hsiehchen

**Affiliations:** Department of Internal Medicine, University of Texas Southwestern Medical Center, Dallas, TX 75390, USA; University of Texas Southwestern Medical School, Dallas, TX 75390, USA; Division of Hematology and Oncology, Department of Internal Medicine, University of Texas Southwestern Medical Center, Dallas, TX 75390, USA

## Abstract

Inactivating mutations in the exonuclease domain of *POLE* induce somatic hypermutation resulting in a high tumor mutation burden (TMB) and are associated with immune checkpoint inhibitor (ICI) benefit. *POLE* mutations outside the exonuclease domain predicted to be deleterious are also observed in cancers, but it is unknown whether they are similarly associated with response to ICIs. We present a patient with hepatocellular carcinoma with a rare POLE mutation (V1368M) outside the exonuclease-domain predicted to be deleterious, a low TMB (1 mut/Mb), and microsatellite stability, who demonstrated an exceptional response to pembrolizumab. To support the generalizability of this finding, an analysis of 1278 patients with advanced cancers harboring low or intermediate TMB treated with ICIs showed that missense non-exonuclease domain *POLE* mutations were associated with greater overall survival. In contrast, among patients with advanced cancers without ICI exposure, *POLE* mutations were not associated with overall survival. These results demonstrate that a subset of missense *POLE* mutations may represent predictive biomarkers independent of TMB. Pathogenic *POLE* mutations outside the exonuclease domain may result in altered functions beyond DNA replication and proofreading which render cancers sensitive to ICIs.

## Teaser

This article presents the case of a patient with hepatocellular carcinoma with a rare POLE mutation outside the exonuclease domain, who demonstrated an exceptional response to pembrolizumab.

**Key Points**

- Deleterious *POLE* mutations outside the exonuclease domain are rare and not associated with high tumor mutation burden.
- The presence of *POLE* mutations outside the exonuclease domain may predict benefit from immune checkpoint inhibitors.

DNA polymerase epsilon (Pol ε) is responsible for replication of the leading strand and proofreading of mismatched bases.^1^ Pol ε may also participate in multiple DNA repair pathways, cell cycle checkpoints, and the maintenance of heterochromatin.^2^ The catalytic subunit of Pol ε is encoded by *POLE*, which contains an exonuclease (exo-) domain responsible for proofreading and a polymerase (pol-) domain. Inactivating mutations in the *POLE* exo-domain are associated with an ultramutated phenotype attributed to error-prone replication and are observed in multiple cancer types including colorectal, endometrial, gastric, breast and brain cancers.^3^

Given the association between *POLE* exo-domain mutations and high tumor mutation burden (TMB), *POLE* mutant cancers are hypothesized to be sensitive to immune checkpoint inhibitors (ICIs).^4^ Indeed, several case studies report deep and durable responses to ICIs among *POLE* exo-domain mutant cancers from diverse tissues.^5,6^ Universal features of these cancers are extremely high mutation frequencies ranging from 117 to 217 mut/Mb.^5,6^ Exceptional responses associated with *POLE* mutations have therefore been attributed to the fact that affected cancers harbor high TMB resulting in an elevated neoantigen load. To challenge this paradigm, we present a patient with hepatocellular carcinoma (HCC) with a rare deleterious *POLE* mutation outside the exo-domain with a low TMB of 1 mut/Mb and microsatellite stability (MSS) who demonstrated an exceptional response to pembrolizumab. To support the generalizability of this finding, an analysis of 1271 patients with advanced cancers harboring low or intermediate TMB treated with ICIs demonstrated that missense non-exo-domain *POLE* mutations were associated with greater overall survival. Thus, deleterious *POLE* mutations outside the exo-domain are not associated with high TMB but may represent predictive markers of ICI benefit.

## Patient Story

A 61-year-old man with a medical history of non-insulin-dependent diabetes mellitus and hepatitis C cirrhosis complicated by variceal bleeding was initially found to have 2 hepatic lesions on a screening ultrasound. Subsequent contrast-enhanced magnetic resonance imaging showed a 2.9-cm right hepatic dome and a 1.5-cm left lobe Liver Imaging Reporting and Data System LR-5 lesion. The patient underwent a partial hepatectomy of the segment 2 lesion with pathology showing a moderately differentiated HCC and microwave ablation of the segment 7 lesion. Follow-up imaging showed a new 3.5 cm segment 8 LR-5 lesion which was treated with stereotactic body radiation therapy. The patient had further recurrence of 3 separate LR-5 lesions over a span of 23 months which were treated with transarterial chemoembolization and microwave ablation. Approximately 14 months after his last loco-regional treatment, surveillance imaging showed new masses including a 1.7-cm LR-5 lesion in segment 7, a 1.5-cm LR-5 lesion in segment 5, and multiple LR-4 lesions. Pathologic evaluation of an LR-5 lesion confirmed the diagnosis of moderately differentiated HCC. Given his multiple recurrences after surgical and loco-regional modalities and multi-focal intrahepatic disease, the patient was referred for systemic treatment.

## Molecular Tumor Board

**Figure 1.**
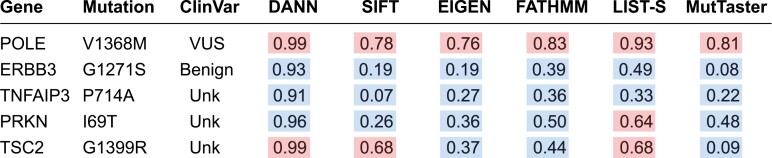
Molecular characteristics of a hepatocellular carcinoma with an exceptional response to pembrolizumab. Missense mutations captured by next generation sequencing of tumor tissue were analyzed by prediction algorithms to determine the likelihood of pathogenic (red) or benign (blue) mutations. Computed rank scores by each algorithm for individual mutations are provided but are not directly comparable across algorithms. VUS, variant of unknown significance. Unk, unknown.

Tumor molecular profiling of the patient’s recent tumor specimen was performed using the FoundationOne CDx assay, a next generation sequencing-based assay analyzing 324 cancer-associated genes. A missense mutation was reported in *POLE* (4102G>A) resulting in a V1368M mutant with a variant allele frequency of 44%. This variant is currently considered a variant of unknown significance in ClinVar. Additional missense mutations were identified in *ERBB3*, *TNFAIP3*, *PRKN*, and *TSC2* (Fig. 1). The cancer was also microsatellite stable and had a TMB of 1 mut/Mb. Given that the POLE mutation was outside of exons 9-14 where hotspot mutations in the exo-domain occur, we used 6 distinct prediction algorithms to confirm that the POLE V1368M mutant is pathogenic (Fig. 1). We performed similar analyses for other missense alterations in the cancer which demonstrated that the remaining mutations were likely benign or tolerated with the exception of the TSC2 G1399R mutation (Fig. 1). An analysis of all cases in cbioportal.org and the ICGC data portal revealed 4 additional cancers (1 gastric cancer, 1 non–small cell lung cancer, 1 glioblastoma, and 1 colorectal cancer) that harbored the POLE V1368M mutation. Based on a literature review and search on ClinVar, germline POLE V1368M mutations have also been observed in at least 2 cases of familial colon cancer.^7^ In contrast, a review of the same genomic resources showed that the TSC2 G1399R mutation has not been previously reported.

**Figure 2.**
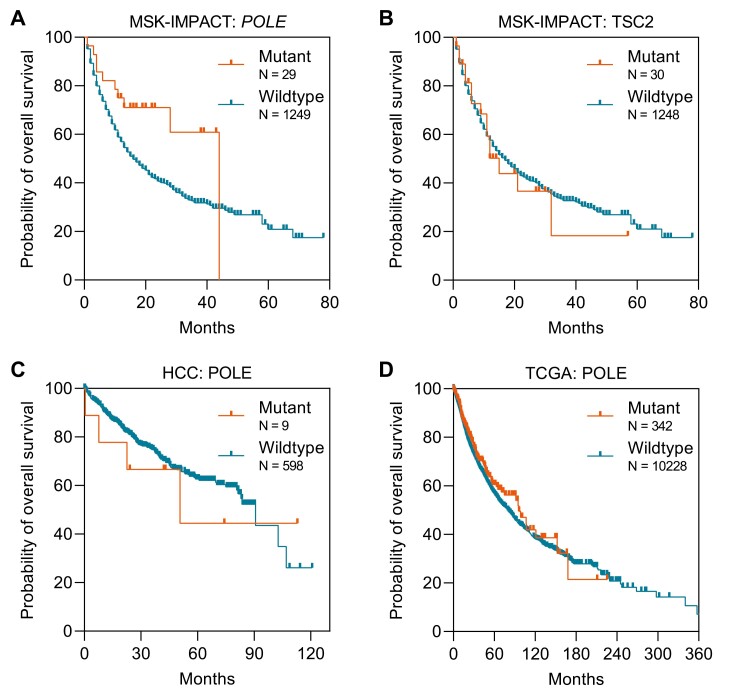
Prognostic and predictive value of *POLE* mutations. (**A**) Kaplan-Meier curves of overall survival for 1271 patients with advanced solid cancers treated with ICIs stratified by the presence of missense *POLE* mutations showed a statistical difference in survival (44.0 vs 16.0 months, log-rank test, *P* = .02). (**B**) Kaplan-Meier curves of overall survival for 1271 patients with advanced solid cancers treated with ICIs stratified by the presence of missense *TSC2* mutations showed no difference in survival (15.0 vs 17.0 months, log-rank test, *P* = .99). (C) Kaplan-Meier curves of overall survival for 1070 patients with HCC stratified by the presence of missense *POLE* mutations showed no difference in survival (50.7 vs 90.6 months, log-rank test, *P* = .68). (D) Kaplan-Meier curves of overall survival for 10 719 patients from The Cancer Genome Atlas stratified by the presence of missense *POLE* mutations showed no difference in survival (96.2 vs 80.0 months, log-rank test, *P* = .16).

We sought to determine whether missense non-exo domain *POLE* mutations are predictive of ICI benefit among patients without high TMB. Given the rarity of *POLE* mutations in HCC and the lack of an existing genomic dataset to analyze ICI-treated HCC, we analyzed a previously described cohort of ICI-treated patients with solid tumors that underwent targeted next-generation sequencing (MSK-IMPACT).^8^ Due to the varying mutation rate between cancer types, we categorized TMB-high cancers as those having a TMB in the highest 20th percentile in each histology because this definition which was previously demonstrated to be associated with overall survival in the MSK-IMPACT cohort.^8^ Among 1278 patients with non-TMB-high cancers treated with ICIs, missense *POLE* mutations outside the exo-domain were associated with a significantly prolonged median overall survival (44.0 vs 16.0 months, *P* = .02; Fig. 2A). In contrast, there were no survival differences when patients were stratified by missense *TSC2* mutations (15.0 vs 17.0 months, *P* = .99; Fig. 2B).

To determine whether missense non-exo-domain *POLE* mutations may represent a favorable prognostic marker independent of treatment context, we analyzed 1070 patients with HCC in cbiportal.org who did not receive ICIs. *POLE* mutant HCC were not associated with a significant difference in median overall survival (50.7 vs 90.6 months, *P* = .68) compared with *POLE* wild-type HCC (Fig. 2C). Of note, no exo-domain mutations were identified in HCC, and the small sample size of patients with HCC harboring *POLE* mutations (*N* = 9) may limit the statistical power to detect a difference in survival. A similar analysis of 10 719 patients in The Cancer Genome Atlas spanning 30 solid cancer types similarly showed no association between missense *POLE* mutations and overall survival (*P* = .16; Fig. 2D). Collectively, these results indicate that missense *POLE* mutations are not HCC-specific or cancer agnostic prognostic biomarkers.

## Patient Update

**Figure 3.**
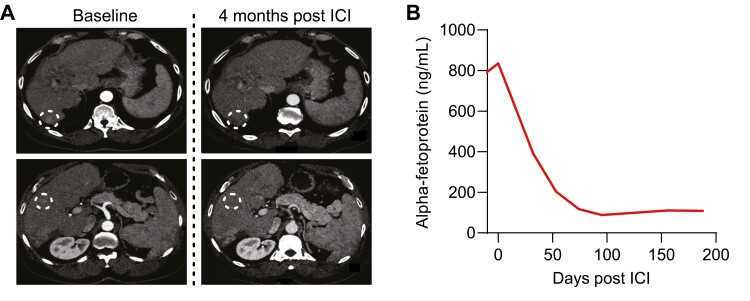
Exceptional treatment response to pembrolizumab. (**A**) Triple phase computed tomography of the abdomen and pelvis using a liver protocol was performed at baseline and 4 months after initiation of pembrolizumab. Each row indicates an axial slice at approximately the same anatomical level across both time points to portray individual HCC lesions (indicated by the dotted white circle). (**B**) α-Fetoprotein levels were serially measured before and after initiation of pembrolizumab.

Although the patient had Child-Pugh A liver disease, monotherapy with pembrolizumab was initiated rather than any anti-VEGF agents given the patient’s history of variceal bleeding. By the fourth week of treatment, α-fetoprotein (AFP) levels had declined from a peak level of 835 ng/mL immediately prior to the initiation of therapy to 392 ng/mL. Radiographic evaluation after 4 months of treatment demonstrated a complete response, which was consistent with further declines in AFP levels (Fig. 3A, B). The patient experienced no adverse events attributed to immune-related toxicities and remains on therapy 8 months after starting pembrolizumab.

## Discussion

As far as we are aware, this report is the first to describe a patient harboring a low TMB, MSS, and a deleterious non-exo-domain *POLE* mutation who experienced a complete response to pembrolizumab which has been sustained to the time of this report (6-months post treatment). While ICIs are FDA approved in the first- and second-line setting for HCC, we believe that the patient’s response is remarkable because rates of complete response reported in the KEYNOTE-224 trial testing pembrolizumab monotherapy in advanced HCC was only 1%. In addition, the low TMB and MSS status of the patient’s cancer would have predicted limited benefit with ICIs.

Multiple lines of evidence support the pathogenicity of the POLE V1368M mutation including consistent predictions from diverse algorithms indicating disrupted protein function or structure. In addition, we identified POLE V1368M somatic mutations in other cancer types and germline mutations in 2 separate patients associated with familial colon cancer syndromes. It is notable that the variant allele frequency of POLE V1368M is near 50%, suggesting that this alteration may represent a germline mutation in the patient. The FoundationOne CDx assay only assesses tumor tissue, so the patient was referred to our genetics clinic but he declined germline testing.

Functional interrogation of the POLE V1368M mutation is needed to clarify its role in mediating the response of cancers to ICIs. Our results suggest that while non-exo-domain mutations do not cause hypermutation, they may enhance immunogenicity through mechanisms independent of neoantigens or represent surrogate markers of other molecular features that drive ICI sensitivity. Non-canonical functions beyond DNA replication and proofreading have been previously proposed for Pol ε, but whether cancer-associated POLE mutations outside the exo-domain may impact such functions remains to be investigated.^9^

We leveraged multiple large genomic datasets to show that missense *POLE* mutations outside of the exo-domain are detected in a non-trivial proportion of cancers and are not HCC or cancer-agnostic prognostic markers. In addition, our analysis of a cohort of ICI-treated patients indicates that a subset of *POLE* mutations outside of the exo-domain may predict ICI benefit among cancers with low or intermediate TMB. In contrast, missense *TSC2* mutations were not associated with ICI benefit in our analysis, suggesting that our patient’s exceptional response to pembrolizumab cannot be attributed to the TSC2 G1399R mutation. Consistent with our findings, preliminary findings in a phase II trial enrolling patients with proficient DNA repair and a missense *POLE* mutation (NCT03012581) showed that 4 of 8 patients (50%) with an exo-domain mutation had a partial response to nivolumab, and 2 of 3 patients (66%) with variants of unknown significance outside the exo-domain had an objective response. In contrast, 0 of 5 patients with *POLE* mutations predicted to be non-pathogenic had a response. Given that many *POLE* mutations observed in cancers are considered passenger alterations, additional investigations are needed to precisely characterize *POLE* mutations that enhance the immunogenicity of cancers and to elucidate whether defective replicative function in POLE is necessary or sufficient for ICI response.

No biomarkers to date are clinically used to guide patient selection for the treatment HCC with ICIs. As first-line treatment options for HCC include oral tyrosine kinase inhibitors, suspected pathogenic non-exo-domain *POLE* mutations may indicate diseases where ICI treatment should be considered. Our results also suggest that non-exo-domain *POLE* mutations may indicate benefit from ICIs in additional cancer types independent of TMB, but this requires prospective validation in larger studies.

## Data Availability

Genomic data sets utilized in this study are publicly accessible on cbioportal.org.
